# We need support! A Delphi study about desirable support during the first year in the emergency medical service

**DOI:** 10.1186/s13049-017-0434-5

**Published:** 2017-09-06

**Authors:** Anna Hörberg, Maria Jirwe, Susanne Kalén, Veronica Vicente, Veronica Lindström

**Affiliations:** 1 Karolinska Institutet, Department of Clinical Science and Education, Södersjukhuset, Academic EMS, Stockholm, Sweden; 2 Department of Neurobiology Care Sciences and Society, Division of Nursing, Stockholm, Sweden; 3 Karolinska Institutet, Department of Clinical Sciences and Education, Södersjukhuset, Stockholm City Council, Stockholm, Sweden; 4 The Ambulance Medical Service in Stockholm (AISAB) Sweden, Karolinska Institutet, Department of Clinical Sciences and Education, Södersjukhuset, Academic EMS, Stockholm, Sweden; 5 Department of Neurobiology Care Sciences and Society, Division of Nursing, Academic EMS, Stockholm, Sweden

**Keywords:** Emergency medical service, Professionals, Professional development, Support

## Abstract

**Background:**

New and inexperienced emergency medical service (EMS) professionals lack important experience. To prevent medical errors and improve retention there is an urgent need to identify ways to support new professionals during their first year in the EMS.

**Methods:**

A purposeful sample and snowball technique was used and generated a panel of 32 registered nurses with 12–48 months of EMS experience. A Delphi technique in four rounds was used. Telephone interviews were undertaken in round one to identify what desirable support professionals new to the EMS desire during their first year. Content analysis of the transcribed interviews yielded items which were developed into a questionnaire. The experts graded each item in terms of perceived importance on a 5-graded likert scale. Consensus level was set at 75%. Items which reached consensus were removed from questionnaires used in subsequent rounds.

**Results:**

Desirable support was categorized into eight areas: Support from practical skills exercises, support from theoretical knowledge, support from experiences based knowledge, theoretical support, support from an introduction period, support from colleagues and work environment, support from management and organization and other support. The experts agree on the level of importance on 64 of a total of 70 items regarding desirable support. One item was considered not important, graded 1 or 2, 63 items were considered important, graded 4 or 5.

**Conclusion:**

Even with extensive formal competence the EMS context poses challenges where a wide variety of desirable forms of support is needed. Support structures should address both personal and professional levels and be EMS context oriented.

## Introduction

Experience has been suggested to be one of the most valuable tools for handling the wide variety of unpredictable situations that emergency medical service (EMS) professionals around the world may encounter [1, 2]. New and inexperienced EMS professionals lack this experience.

To prevent medical errors and improve retention there is an urgent need to identify ways to support new professionals during their first year in the EMS.

Without knowledge about what the EMS professionals themselves would desire during their first year, well-intentional support strategies risk being unsuccessful.

## Background

The required level of competence needed in the EMS differs around the world [3, 4]. In Sweden where this study was conducted an ambulance is staffed by at least one registered nurse (RN) and an emergency medical technician [5].

However, the patients and challenges that EMS professionals meet are the same worldwide [6–9].

EMS professionals are exposed to the full extent of human emotions, injuries and suffering in a wide variety of unfamiliar, unpredictable and potentially dangerous environments [5, 10, 11].

Competence can be defined as “the ability to do something successfully” https://en.oxforddictionaries.com/definition/competence and will be used in this study when referring to the skills and knowledge needed to care for the great variety of patients encountered in EMS. Today many EMS professionals’ competence is assessed through the use of techniques such as simulation-based exercises. However, a professional’s competence and readiness for the independent and clinical work in the EMS can be complex for educators to assess [12]. Prior experience has been described as one of the most important tools for handling the many different situations a professional may encounter [1].

A professional practice like the EMS is a far from ideal clinical work environment. Peer support is limited since the professionals work with only one partner. In most countries, a physician is available only via telephone, and sometimes that physician does not work in the EMS [2, 13]. A new EMS professional needs to be able to work independently and make quick critical decisions from day one.

The first year in professional practice is associated with insecurity, feelings of stress, self-doubt and inadequacy [14, 15]. Being unsupported, new and inexperienced may have significant consequences for patient safety [16, 17]. Inexperience has been associated with a higher degree of medical errors, delay in care and errors in critical thinking [18].

Even though simulation is a successful tool for the training and development of professional skills [19] it can never be more than a surrogate of reality. Something else, or rather something more might be needed to support the development and maintenance of competence in the EMS during the first year of practice.

In a plethora of support systems suitable for ‘in-hospital’ environments there seems to a lack of knowledge regarding what kind of support is eligible for the ‘out of hospital’ EMS. To provide a basis for discussion and future interventions this study aimed to identify the support desired by new and inexperienced EMS professionals during their first year in the EMS.

## Method

### Study design

The Delphi technique was used to achieve consensus on desirable support during the first year in the EMS in a group of informants considered to be experts on being new. The Delphi is an iterative process characterized by a number of rounds in which questionnaires are sent out until consensus is reached [20]. This Delphi study commenced with interviews and a broad open question, and the classical Delphi technique with four rounds was used [21]. Data was collected during April–September 2016.

### Panel of experts

An expert is defined as a person who is very knowledgeable about a particular area https://en.oxforddictionaries.com/definition/expert

This study was performed in Sweden where an ambulance is staffed with at least one RN or a specialist nurse with a specialist degree and an emergency medical technician [5]. A specialist nurse has a one year post-graduation education degree in a subject such as pre-hospital emergency care or intensive care.

This study involves both RNs and specialist nurses, henceforth referred to as RNs. All RNs were considered experts on the experience of being new and what support they themselves would have desired during the first year in the EMS.

To obtain a wide range of perspectives on desirable support, a purposeful sample and snowball strategy was used [22]. Region directors and personal knowledge were used to identify the initial RNs. All RNs that consented to participate in the study were asked to recommend other possible experts. To avoid influence of personal gain and for the RNs to be able to reflect on their first year, the RNs had to have worked for more than 12 months in the EMS. Furthermore, for the RNs to have reached a competent level [23] and still be able to relate to the first year, an upper limit of three years of experience was set. In total 32 experts agreed to participate (Table 1).

**Table 1:** Demographic information of participants

Demographics		Number (percent)
Total of included experts		32 (100%)
Gender	Male	12 (37.5%)
	Female	20 (62.5%)
Academic degree	Registered nurse	9 (28%)
	Specialist nurse *(Pre-hospital emergency care)*	18 (56%)
	Other specialist nurse	5 (16%)
Geographic region	Urban	16 (50%)
	Sub-urban	10 (31%)
	Rural	6 (19%)
Months of experience *(in the EMS)*	12–24	25 (78%)
	> 25	7 (22%)
Years of RN experience	< 5	11 (34%)
	5–10	15 (47%)
	> 10	6 (19%)

### Round 1:

The first round was an idea generating round that consisted of open-ended sets of questions [24]. For this round, individual telephone interviews were used, comprising three questions:

1. Can you tell me about a situation during your first year in which you did not experience that you could manage the way you would have liked to?
2. What support would you have desired to manage that particular situation?
3. During your first year, what other support, apart from that you just described, would you have desired?

All experts received the questions in advance and interviews took place at times decided by the experts. The interviews were tape-recorded and transcribed verbatim after each interview. The transcribed material was analyzed using the manifest content analysis described by Hsieh and Shannon [25] (Table 2).

**Table 2:** Examples of the content analysis process in Round 1

Interview	Meaning unit	Code	Category	Item
When you need it to just flow, where time is of essence…and…it’s hard to say exactly but you can think of situations where someone dies, gives birth, is severely ill or children…you can do a lot of exercises on those situations…we do practice traffic accidents and stuff like that, but we should have more exercises on the ordinary things… (Expert #1)	Practice situations where time is of the essence and the ordinary things (Expert #1)	Practical exercises (Experts#1, 3, 5, 12, 13, 14, 16, 17, 18, 19, 20, 22, 23, 27, 31)	Support from practical exercises	Practice situations that occur rarely (Experts #1, 5, 12, 13, 14, 16, 17, 19, 22, 23, 31) Practice situations that occur frequently (Experts#1, 3, 5, 14, 17, 18, 19, 20, 27)
To me, it would have been supportive to have some kind of mentor…maybe a mentor that you would come back to and have discussions with and meetings where you could discuss different situations you have experienced with other colleagues or patients…to have someone to bounce things off… (Expert #16)	It would have been supportive to have a mentor to discuss situations that had been encountered with colleagues or patients (Expert #16)	To have a mentor (Experts #2, 5, 6, 7, 8, 9, 12, 14, 16, 17, 19, 21, 24, 25, 26, 27, 29, 30)	Support from colleagues and work environment	Have a mentor that supports your personal development (Experts #2, 5, 6, 7, 9, 12, 14, 16, 17, 19, 21, 25, 26, 27, 29, 30)

Round 1 resulted in eight categories concerning desirable support and a total of 62 statements, henceforth referred to as items, about desirable support.

### Round 2:

The items generated in round 1 were used to construct a questionnaire. To enhance validity the original questionnaire was piloted in a group of seven persons who did not participate in the main study [22]. The pilot group consisted of two RNs new to the EMS, two researchers, and three experienced RNs with a special interest in education. The final questionnaire in round 2 consisted of eight categories with a total of 65 items. The experts were asked to grade the importance of each item using a five-point Likert scale ranging from ‘not at all important’ (score 1) to ‘very important’ (score 5). In this round, the questionnaire also included open questions where the experts were asked to add additional items if they considered anything to be missing. The questionnaire was distributed to the 32 experts. Two reminder emails were sent during a three-week period, resulting in a 100% response rate.

As suggested in other studies [26, 27], for analytical purposes the scale was tricotomized to a three-point scale before determining if consensus had been reached. This means that the two lower responses “1–2” represented not important, “3” represented neutral and two upper responses “4–5” represented important. This was done on the assumption that people tend to grade either the highest/lowest or the next highest/lowest, arguing that the item is ‘as good as it gets’ or ‘it is important but it can always be better’ [26]. The level of consensus was predetermined and set at 75%. An item was considered to have reached consensus when 75% (24/32) or more experts agreed on any of the tricotomized scale responses. When calculating the frequencies using the tricotomized scale for each item in round 2, 51 items reached consensus and the experts suggested five new items (Table 3).

**Table 3:** Delphi flow chart of the four rounds

	Round 1	→	Round 2	→	Round 3	→	Round 4
Number of participants	32		32		32		31
Response rate	100%		100%		97%		100%
Drop-out	0		0		1		0
Round activity	Interviews analyzed by manifest content analysis	→	Questionnaire with 65 items	→	Questionnaire with 19 items (14 not reaching consensus in round 2 + 5 new)	→	Questionnaire with 9 items
			↓		↓		↓
			51 items with consensus reached		10 items with consensus reached		3 items with consensus reached

### Round 3:

A new questionnaire was constructed comprising 19 items; 14 items that did not reach consensus in round 2 and five new generated in the open-ended questions in round 2 (Table 3.). Feedback containing the median value and the experts’ individual response on each of the 14 items from round 2 was provided in a personal PDF document to each expert. Median value was used to show how a majority had rated each item [21]. A technical problem occurred during the process leading to a risk of individual feedback in this round being inaccurate. The median values were unaffected and accurate. The experts were informed about this risk and asked to take this into consideration when reconsidering their grading in round 3.

The questionnaire was distributed to the 32 experts and two reminder emails were sent during a four-week period resulting in 97% response rate. One expert did not complete the questionnaire.

The responses were analyzed as in round 2 and consensus was reached on a further 10 items.

### Round 4:

A final questionnaire was constructed with the remaining nine items from round 3 (Table 3.). This was distributed with a PDF with feedback as previous rounds. The questionnaire was distributed to the 31 experts that completed round 3. One reminder email was sent and in 10 days the response rate was 100%. The final round generated another three items on which consensus was reached and six where it was not.

### Ethical considerations

All the experts were informed about the study both in written and oral form, and participation was voluntary. Confidentiality was guaranteed and the experts were informed that they could leave the study at any time.

Because of the iterative nature of the Delphi technique, true anonymity cannot be guaranteed and the term quasi-anonymity is more often used. The first author (AH) knew the identity of the experts, and to provide individual feedback, AH also had to be able to identify experts’ individual responses. No personal data was included in the report of the results and AH was the only author who knew the identity of the experts. The experts were informed about this and all gave their consent.

The need for ethics approval was waived by the Regional Ethical Board in Stockholm (Diary number 2015/87–31/5).

## Results

All items that reached consensus presented in mean values, standard deviation (SD) and in what round consensus was reached are presented in Table 4. Items for which consensus was not reached are presented in Table 5.

**Table 4:** Results from Delphi rounds

	Mean value	Standard deviation SD	Consensus reached in round
Support from practical skills exercises			
Practice methods to get a structured way to work (e.g. according to the ABCDE-principle)	4,8	0,7	2
Practice ways to lead the work at the scene of an accident	4,5	0,8	2
Get a structured run-through of the medications used in the EMS	4.5	0.8	3
Practice through simulation	4,4	0,7	2
Practice with the radio communication equipment	4,4	0,9	2
Practice with the medical equipment in the ambulance	4,4	0,9	2
Practice techniques for immobilization	4,4	0,9	2
Practice in collaboration with police and rescue service	4,4	1,0	2
Practice situations that occur rarely	4,3	0,8	2
Practice techniques for removing people from vehicles	4,3	0,9	2
Have practical skills tests	4.3	1.0	2
Practice situations that occur frequently	4,3	1,1	2
Driving and parking exercises	4,1	1,1	2
Practice techniques to maneuver the stretcher	4.0	0.9	3
Support from theoretical knowledge			
Have access to lectures on medical conditions in children	4,5	0,7	2
Get a structured run-through of the EMS medical guidelines	4,5	0,8	2
Get access to concept educations such as AMLS, PHTLS, PS, PEPP	4,4	0,8	2
Have access to lectures on medical conditions in adults	4,3	0,8	2
Have written tests on theoretical knowledge	4,3	0,9	2
Have access to lectures on how to lead the work at the scene of an accident	4,2	0.8	3
Have access to lectures on child birth	4,2	0,9	2
Support for experience-based knowledge			
Get feedback on the own actions from the receiving unit	4,8	0,4	2
Participate in courses along with experienced colleagues	4,7	0,6	2
Participate in group discussions about authentic patient situations	4,7	0,5	2
Participate in group discussions about ethics	4,5	0,8	2
Participate in group discussions about threats and violence	4.1	0.9	3
Theoretical support			
Have access to applicable medical guidelines	4,8	0,4	2
Have access to internet-based instruction films on the ambulance’s technical equipment	4.3	0.6	3
Have access to written guidelines on when and how to contact a physician	4,2	0,8	2
Have access to written guidelines about how to report deviations	4.1	0.9	3
Have access to instruction films about how to realign a fracture	4.0	0.8	3
Support from an introduction period			
Get regular feedback on the own development during an introduction period	4,9	0,2	2
Have a structured introduction period	4,9	0,3	2
Have an individually fitted introduction period	4,7	0,7	2
Have a supervisor with formal supervisor competence	4,4	0,8	2
Work with the same supervisor during the introduction period	4,0	0.7	3
Work with the same ambulance team (supervisor and his/her colleague) during the introduction period	3.7	0.8	4
Support from colleagues and work environment			
Get peer support debriefing in extreme situations	5,0	0,2	2
Have a trustworthy colleague	4,9	0,4	2
Have an experienced colleague	4,8	0,5	2
Be respected and accepted by the colleagues at the ambulance station	4,8	0,5	2
There is an open climate at the ambulance station	4,8	0,6	2
Have one person in the organization to contact with logistics questions during off-hour	4.6	0.6	2
Have a mentor to contact about routines	4,5	0.8	3
Have a mentor to support professional development	4,3	0,8	2
Have a mentor to talk to about conflicts	4,3	1,0	2
Have a mentor to contact about practical issues	4,2	0,8	2
Have a mentor to support personal development	4,0	0,9	2
Work with another RN	3.9	0.9	4
Support from management and organization			
Trust in the ambulance station manager	4.8	0.4	2
Have confidence in the organization	4,8	0,4	2
The organization is characterized by professionalism	4,8	0,6	2
Get feedback on the own professional development from the ambulance station manager	4,7	0,6	2
The organization accepts that new professionals need more time to perform patient assessments	4,6	0,6	2
The organization provides time for professional development activities	4,6	0,7	2
The organization is characterized by equally	4,6	0,7	2
The organization has clear competence descriptions of what is expected of each role in the team	4,5	0,8	2
The organization is characterized by ethical considerations	4,5	0,7	2
The dispatch center accepts that new professionals need more time to perform patient assessments	4,4	0,7	2
Trust in the organization director	4,3	1,0	2
Get feedback on the own professional development from the organization director	4.1	0.9	3
Other support			
Being exempt from introducing new colleagues	4,6	0,9	2
Have access to an interpreter service	4.1	0.7	4
Being exempt from life-threatening assignments with the highest level of priority	1,4	0,7	2

**Table 5:** Items for which consensus was not reached

	Mean value	Standard deviation SD
Support from theoretical knowledge		
Get access to lectures about psychiatric conditions	3.7	0.7
Be able to visit and auscultate at different intra-hospital wards	3.6	1.0
Theoretical support		
Have access to written ethical guidelines	3.5	0.8
Have access to written guidelines regarding how to manage conflicts	3.5	0.8
Support from colleagues and work environment		
Work with the same colleague during the first year	2.9	0.9
Other support		
Receiving an extra unit when being given life-threatening assignments with the highest level of priority	2.4	0.8

### Round 1:

The analysis of the telephone interviews and the pilot round generated 65 items that were grouped into eight categories describing desirable support during the first year in the ambulance service:

*Support from practical skills exercises* encompasses practical skills training, such as practicing with the ambulance equipment, simulation exercises and collaboration exercises. Some of the RNs also described a practical skill test as desirable support.

*Support from theoretical knowledge* encompasses lectures on medical conditions in adults and children, childbirth, lectures on how to structure work in the ambulance and how to use the medical guidelines. The RNs also described theoretical knowledge test to be supportive.

*Support for experience-based knowledge*. This category comprises support derived from experienced colleagues, such as being able to reflect with the colleagues about different patient situations and ethical dilemmas and to get feedback from the receiving units.

*Theoretical support,* the RNs wanted to have different support devices such as written guidelines for medical conditions, ethical dilemmas, when and how to contact a physician, how to deal with conflicts and how to report deviations.

*Support from an introduction period* encompasses desirable support specifically during the first weeks or months in the EMS. The RNs wanted to have an individually fitted and structured introduction period where they could work as third person with colleagues that were educated supervisors. Some of the RNs wanted to work with the same supervisor during the first weeks or months and some wanted to work with the same EMS team, i.e. both the supervisor and his or her partner. To received feedback on the own development was also desirable during this time.

*Support from colleagues and work environment.* This category comprises desirable attributes of the colleague, such as being experienced, trustable and being a nurse. It also comprises support from a mentor. This was mentioned by more than half of the RNs. Others talked about having a trusted colleague or ‘someone’ to talk to. It was also desired that there was an open climate and that the colleagues respected and accepted the new professionals. All RNs that described debriefing in situations of crisis said that this was something that the own organization already provided.

*Support from management and organization* encompasses the role of the managers and factors desirable to underpin the organization. Time was described as a desirable support in terms of being able to use more time to assess patients, and that the EMS organization or agency allocated time for professional development activities. This category also included the desire for a clear description of what the organization and managers expected from the EMS professionals.

*Other support,* this category comprises the items that did not fit into any of the other categories, such as receiving an extra unit when being given life-threatening assignments with the highest level of priority, being exempted from life-threatening assignments with the highest level of priority, and not having to supervise new colleagues themselves during the first year.

### Round 2:

Fifty-one of the 65 items reached the predetermined consensus level of 75%. Consensus was reached on all items in the category *support for experience-based knowledge* and were all considered very important (graded 4 or 5). In contrast, consensus was reached on only two items in the category *theoretical support*. Fifty of the 51 items on which consensus was reached in this round were considered very important (graded 4 or 5). In the category *other support*, the RNs agreed that being exempt from life-threatening assignments with the highest level of priority was ‘not important’ (graded 1 or 2).

In this round five additional items were created out of the free text questions and added to the questionnaire for round 3.

### Round 3:

During this round, consensus was reached on 10 more items. The category *support from practical skills exercise* consisted of 13 items and was the category with most items. In this round consensus was reached on the last of the remaining items in this category. Consensus was also reached on the last two items in the category *support from management and organization.* All items that for which consensus was reached in this round were considered to be very important (graded 4 or 5).

### Round 4:

Consensus was reached on a further three items in the final round.

In total, consensus was reached on 64 out of 70 items after four rounds. One of these 64 items ‘being exempt from life-threatening assignments with the highest level of priority’ was considered to be not important, mean 1.4 (SD 0.7), and the rest were considered important with mean values between 3.7–5.0 (SD 0.2–1.0). In the categories, *support from practical skills exercises, support for experience-based knowledge, support from an introduction period* and *support from management and organization* consensus was reached on all items. The categories *theoretical support* and *support from theoretical knowledge* both comprised two items on which consensus was not reached. In the remaining categories, *support from colleagues and work environment* and *other support* consensus was reached on all but one item.

The mean values for the six items for which consensus was not reached ranged between 2.4–3.7 (SD 0.7–1.0) these items are presented in Table 5.

## Discussion

The Delphi technique was proved to be a successful technique for gaining consensus in a group of experts about desirable support for new and inexperienced EMS professionals during the first year in the EMS. The experts in this study agreed on the importance and non-importance on 64 of 70 items concerning desirable support.

It is important to note that consensus on an item does not mean that the right answer or opinion has been found. The results carried out by using a Delphi technique help identify areas that a group of experts consider important and should be used as a basis for further discussion [22].

The desirable support revealed by this study is related to both personal development e.g. having a mentor (mean: 4.5–4.0) and the development of professional competence e.g. attending different theoretical lectures (mean: 4.5–4.2) and practical exercises (mean: 4.8–4.0).

It was considered desirable to have a mentor or someone to talk to about routines (mean: 4.5), practical questions (mean: 4.2), and conflicts (mean: 4.3), and to get confirmation on professional (mean: 4.3) and personal (mean: 4.0) development. Mentorship has been considered a key element of health care practice for several years [28, 29]. Even though a need for and prospective benefits of mentorship have been elucidated in the EMS [2, 30, 31], to our knowledge little is known about how a mentorship program in the EMS could be constructed. Effective mentorship can create conditions for the development of a wide range of professional competences such as collaboration and reflectiveness, improved communication skills and development of inter professional relationships, and can also improve patient assessment [29, 32]. EMS professionals are exposed to challenging and sometimes traumatic situations, and posttraumatic stress disorder is not uncommon [33–35]. Mentorship has also been shown to provide emotional support where the mentor can act as a free-zone, being available on an informal basis to talk, and provide advice, acceptance and friendship, which helps the new professionals balance work and life issues [29, 32]. This study indicates that emotional support in a crisis is considered extremely important. This is shown by the fact that the item regarding ‘peer support debriefing in extreme situations’ was the only item with a mean value of 5.0 (SD 0.2). The consensus reached on items regarding mentorship in this study will provide an important basis for further discussions and for the development of mentorship programs in the EMS.

Most of the desirable forms of support regarding practical skills training discussed in this study were EMS-oriented such as radio communication and collaboration with the rescue service and police. Even though the experts in this study were RNs, none of the practical skills items concerned nursing specific skills. This outlines the importance and meaning of the health care context rather than the level of formal competence when designing support structures. What is new and challenging in the EMS are the EMS-specific issues. The EMS has been defined as a context in which the professionals work alone, having the sole responsibility for patient care in an environment that is highly varied and unpredictable and where support from a large team of colleagues is lacking [36]. Support structures based on this study may be well suited even for other contexts with similar prerequisites, for example the police, flying doctors or nurses working in primary care in rural areas.

Furthermore, research indicates that more practical skills training in the EMS is required and a need for increased simulation-based training has been suggested [19].

Many of the items where consensus was reached related to acute situations or serious conditions such as trauma or traffic accidents, or were related to childbirth or caring for sick and injured children.

EMS professionals often report caring for children or assignments involving childbirth as major stress factors which cause them to experience insecurity [37, 38]. In many countries, there are postgraduate nurse or nurse practitioner programs in both childcare and midwifery, indicating that these care situations demand extended education. Since working with childcare and childbirth require an additional post-graduation education, not many of the EMS professionals may have prior experience of working in these fields. Prior experience is considered one of the most important tools for handling challenging situations [1, 39]. The importance of experience in the EMS is further stressed in this study by the category *support from experience-based knowledge,* where consensus was reached on all items and the item ‘having an experienced colleague’ reached a mean value of 4.8.

Psychiatric patients are common and are considered a challenging patient category in the EMS as well [39, 40]. However, the only item regarding psychiatric patients, ‘lectures about psychiatric conditions’ (mean = 3.7 SD 0.7) did not reach consensus. This may be related to difficulties in defining psychiatric care in the EMS.

That the item about psychiatric care did not reach consensus does not necessarily mean that support with this type of patient category is not desired. Experienced EMS professionals describe psychiatric patients as challenging, and more education and supportive measures have been called for [39]. New and inexperienced professionals may focus more on support aiming to manage direct life-threatening situations, and it is only after a few years of practice that the full complexity of EMS may be grasped. It can take years before the stage of proficiency is reached and situations can be seen as whole [23]. This is also illustrated in the desirable support regarding written guidelines and a written competence description. Novice professionals rely on written rules and guidelines to direct their actions [23]. According to Duchscher [15] in the first period of time at a new workplace much effort is devoted to trying to understand what is expected, and doing it well. The item regarding applicable medical guidelines (mean 4.8) further illuminates the need for contextual adaptation when designing support structures. The guidelines provided in the EMS today have been described as not applicable and need to be written by people with knowledge about the EMS context to avoid implicit use of the guidelines [41]. Contextually-based guidelines would perhaps further support new and inexperienced professionals in the EMS. With an increase use of guidelines patient safety may also be increased.

### Methodological considerations

One strength of the Delphi technique is that it enables reaching an agreement in a group of experts avoiding situations where one panel member dominates the consensus process. However, there are Delphi studies that include physical meetings, arguing that this will benefit clarification of reasons for disagreement [42].

Even though the experts in this study were different in regard to background, gender, age, and geographic areas, they were all nurses and the group was treated as homogenous. Comprising experts of different background and gender provides an expert panel with a variety of viewpoints that may provide relevant input to the Delphi and minimize the risk of bias [20].

This Delphi included 32 experts more experts might have revealed even more desirable support and richer descriptions. No precise sample sizes are advocated for Delphi, although panels between 10 and 50 participants, depending on the purpose of the study, have been recommended [26]. When deciding on panel size, the researcher needs to balance the risk of a low response rate against panel size. If the panel size is too large then the number of generated items could be overwhelming. Creating a personal bond, which is considered important to increase response rates, might be also difficult if panel sizes are too large. In this Delphi study the response rate was high and generated a manageable number of items that were considered conclusive.

There is no clear guidance regarding the appropriate level of consensus in the Delphi literature. The predetermined level of 75% was chosen as it has been recommended and has previously been used [20, 26].

In a Delphi study, individual feedback is provided to encourage an expert panel to be more involved and to increase response rates. In this study, the response rate was high throughout all rounds (100%, 97%, 100%). Only one drop-out occurred in round 2 and with respect for the integrity of the expert who dropped out, the reason for leaving the study was not questioned.

The median values and the five new items were not affected by the technical problem occurring in round 2. Since consensus was reached on another 10 items in round 3 and the response rate stayed high, this indicates that the RNs’ desire to stay involved in the study was not negatively affected by the technical problem. In round 3 the problem had been corrected and individual feedback was provided accurately.

## Conclusion

In the EMS, the required level of competence may differ around the world; however, the patients and challenges are the same. The rather large amount of different support items generated in this study show that even though a group of experts in the EMS have a rather similar level of competence they describe a wide variety of desirable forms of support. This implies that even with extensive formal competence the EMS context poses challenges where formal and structured support is needed. The challenges EMS professionals meet can be extreme and emotional support in these situations is especially important.

The results of this study suggest that support for new and inexperienced professionals should address both professional and personal levels and should be context-oriented.

This study may be used as a basis for further discussions about how to design and implement formal support models in the EMS.

The need for support in the EMS has been stressed before but to our knowledge there are few support structures that have been implemented and evaluated. There is a need for further research to investigate what the obstacles are that have led to this lack of formal support structures in the EMS. When the obstacles to overcome are known, design and implement support for new EMS professionals can commence.
